# Comprehensive clinical evaluation of indirect and direct bonding techniques in orthodontic treatment: a single-centre, open-label, quasi-randomized controlled clinical trial

**DOI:** 10.1093/ejo/cjae036

**Published:** 2024-10-04

**Authors:** Kana Kono, Takashi Murakami, Saori Tanizaki, Noriaki Kawanabe, Atsuro Fujisawa, Masahiro Nakamura, Mitsuhiro Hoshijima, Takashi Izawa, Hiroshi Kamioka

**Affiliations:** Department of Orthodontics, Graduate School of Medicine, Dentistry and Pharmaceutical Sciences, Okayama University, Okayama, Japan; Department of Orthodontics, Okayama University Hospital, Okayama, Japan; Department of Orthodontics, Okayama University Hospital, Okayama, Japan; Department of Orthodontics, Graduate School of Medicine, Dentistry and Pharmaceutical Sciences, Okayama University, Okayama, Japan; Department of Orthodontics, Okayama University Hospital, Okayama, Japan; Department of Orthodontics, Okayama University Hospital, Okayama, Japan; Department of Orthodontics, Graduate School of Medicine, Dentistry and Pharmaceutical Sciences, Okayama University, Okayama, Japan; Department of Orthodontics, Graduate School of Medicine, Dentistry and Pharmaceutical Sciences, Okayama University, Okayama, Japan; Department of Orthodontics, Graduate School of Medicine, Dentistry and Pharmaceutical Sciences, Okayama University, Okayama, Japan

**Keywords:** bonding techniques, orthodontic treatment, orthodontic brackets

## Abstract

**Background:**

Few prospective investigations have compared direct and indirect techniques through comprehensive and detailed clinical evaluations, considering the impact of all factors.

**Objectives:**

This study aimed to compare and evaluate direct and indirect bonding methods at a single institution and to clarify the selection criteria for the bonding method.

**Materials and methods:**

This single-centre, quasi-randomized controlled clinical trial included 153 patients who required fixed orthodontic treatment. They were randomly divided into indirect and direct binding groups by the project lead (K.K.), who was blinded to all clinical data, and performed the allocation using medical record numbers. The chair time for bracket bonding, discomfort during bracket bonding, oral hygiene after bonding, number of bracket failures, number of intentional bracket reattachments, post-treatment occlusal index, and total treatment time were assessed. Outcomes were compared using a two-sample *t*-test or Mann–Whitney *U* test (*P* < .05).

**Results:**

Fifty-eight patients were included in the indirect bonding group (20 male, 38 female; mean age: 20.63 ± 5.69 years) and 66 (14 male, 52 female; mean age: 23.17 ± 8.83 years) in the direct bonding group. Compared to the direct bonding group, the indirect bonding group had shorter chair time (*P* < .001), a shorter total treatment period (*P* < .01), and a better final occlusal relationship (*P* < .001). The number of bracket detachments was higher (*P* < .001) in the indirect bonding group, but the number of intentional reattachments was lower (*P* < .001).

**Conclusion:**

Indirect bonding may improve the efficiency of orthodontic treatment.

**Harms:**

No harm was observed during the study.

**Trial registration number:**

This trial was approved by the Ethics Review Committee of Okayama University (approval number: d10001), UMIN registration number 000022182.

## Introduction

Dental brackets are used in fixed orthodontic treatment to align and straighten permanent teeth and help position them depending on a patient’s malocclusion while also improving dental health. Accurate bracket placement is essential for effective and efficient fixed orthodontic treatment. Improperly positioned brackets make the finishing stage of comprehensive orthodontic treatment more difficult and time-consuming and increase the risk of unpredictable reactions to tooth movement [1].

Orthodontic bracket placement can be performed using two techniques. The first is the direct technique, in which the brackets are directly placed on the enamel surface by the operator, as initially described by Newman in 1965 [2]. The second method is the indirect bonding technique, first described by Silverman *et al*. in 1972 [3]. The indirect bonding technique involves a two-stage procedure. The first stage is performed in the laboratory, where the position of the brackets is determined and these are attached to a plaster model of the patient’s teeth. In the second stage, the brackets in their positions are transferred to the patient’s mouth using a tray and are bonded to the etched enamel surface [4]. With recent advances in digital technology, computer-assisted bracket placement has also been introduced for indirect bonding. This technique uses orthodontic software to virtually indirectly bond the brackets based on an initial intraoral scanning or plaster model taken before bonding [5, 6].

The advantages and disadvantages of direct and indirect bonding techniques have been previously discussed [7–10]. Direct bonding can be easily performed, and the bond strength may be improved because of the close fitting of the bracket base to the tooth surface [11]. However, direct bonding is believed to take longer and is a more challenging procedure for orthodontists than indirect bonding [7, 10]. In contrast, in terms of bracket placement accuracy, many reports have indicated that indirect bonding is superior because it is easier to place brackets on models (better vision and unlimited working time) than on teeth *in vivo* [3, 7, 10]. Improving bracket placement accuracy may reduce the need for subsequent repositioning and even shorten treatment time [1]. However, indirect bonding is more technique sensitive and requires more laboratory procedures and time than direct bonding [12]. Additionally, several cross-sectional and retrospective studies have shown high failure rates for brackets placed indirectly during treatment [11, 13]. On the other hand, weak evidence in a systematic review suggested that the direct and indirect bonding techniques had no significant difference in bracket placement accuracy, oral hygiene status, and bond failure rate, for bonding orthodontic brackets [14]. However, most reports compared and evaluated single factors involved in direct and indirect methods, and few prospective studies have considered the relative effects of all factors. Therefore, this study aimed to evaluate and compare the direct and indirect methods through comprehensive and detailed clinical evaluation at a single institution and to clarify the selection criteria for the bonding method. The chair time, subjective sensation (visual analogue scale, VAS), pre- and post-treatment plaque control record (PCR) values, number of bracket failures, number of intentional bracket reattachments, total treatment period, and occlusal relationship at the end of the treatment (cast-radiograph evaluation, CR-Eval) were evaluated.

## Materials and methods

### Eligibility criteria

All patients who met the inclusion criteria were invited to undergo screening. The main inclusion and exclusion criteria are listed in Supplementary Table 1. Written informed consent was obtained from the patients before the screening or inclusion procedure. Patients who did not consent to participate in the study were excluded. This single-centre, open-label, quasi-randomized controlled clinical trial was conducted in compliance with the principles of the Declaration of Helsinki, and the protocol was approved by the Institutional Review Board of Okayama University Hospital (approval number: d10001, UMIN registration number: 000022182). The protocol has been previously published [15].

### Endpoints

We performed a detailed comparison of indirect and direct bonding techniques. The primary outcome parameters are ‘Total treatment period (months)’. The secondary outcome measures are ‘discomfort at bonding (VAS scores)’, “oral hygiene (PCR [16]) after bonding (%),““post-treatment occlusal index (CR-Eval by the American Board of Orthodontics [17] scores),“ ‘Chair time (min)’, ‘Failure (bracket detachment from a tooth) number’, and ‘number of intentional reattachments’. The null hypothesis was that the efficacy of indirect bonding would not markedly differ from that of direct bonding. All enrolled patients will be followed up for 1–3 years after bonding (Supplementary Fig. 1).

### Treatment methods

#### Sample size calculation

The sample size calculation is based on the primary outcome total treatment times. The sample size was determined using the results from a previous retrospective study that compared the difference in treatment times between direct bonding (*N* = 11; total treatment time: 22.91 ± 4.35 months) and indirect bonding techniques (*N* = 35; total treatment time: 14.23 ± 5.02 months) [18], using Power and Sample Size Calculation software (version 3.1.2; Department of Biostatics, Vanderbilt University, TN, USA). The calculation was based on the number of participants required for a two-sample *t*-test. The minimum clinically important difference was 3.16 months. Considering the occurrence of dropouts, the target sample size was calculated to be 50 per group, based on a significance level of 0.05, a power of 80, and a standard deviation of 5 points in both groups.

#### Intervention

The study commenced in January 2015 and ended in March 2019. Following confirmation of eligibility, patients were randomized to undergo treatment with either the conventional direct bonding technique or the indirect bonding technique. There were 21 participating orthodontists, all of whom were full-time employees of the Department of Orthodontics, Okayama University Hospital, and had already been preliminarily reviewed for their knowledge of basic orthodontia and direct and indirect bonding techniques. Every operator performed both direct and indirect bonding techniques. The orthodontists’ clinical experience ranged from 1 to 6 years.

**Figure 1.**
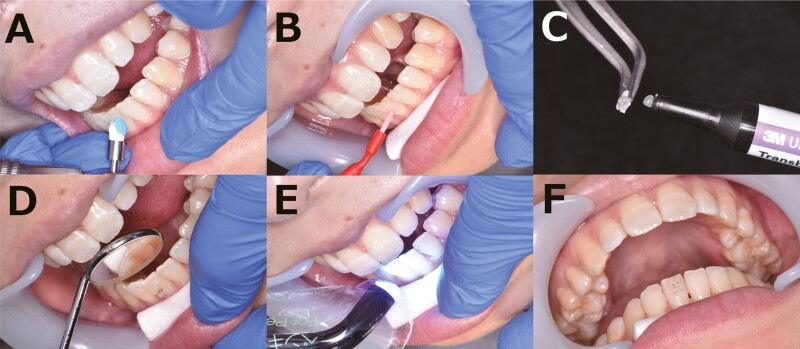
Direct bonding technique. (A) The teeth were cleaned with polishing brush. (B) Etching was applied to the tooth surfaces at the points where the brackets would be attached. (C, D) The brackets were applied directly to the tooth surfaces, and bonded using the Transbond XT Adhesive. (E) After the brackets were placed, photopolymerization was achieved with a curing light device applied. (F) bracket was attached.

**Figure 2.**
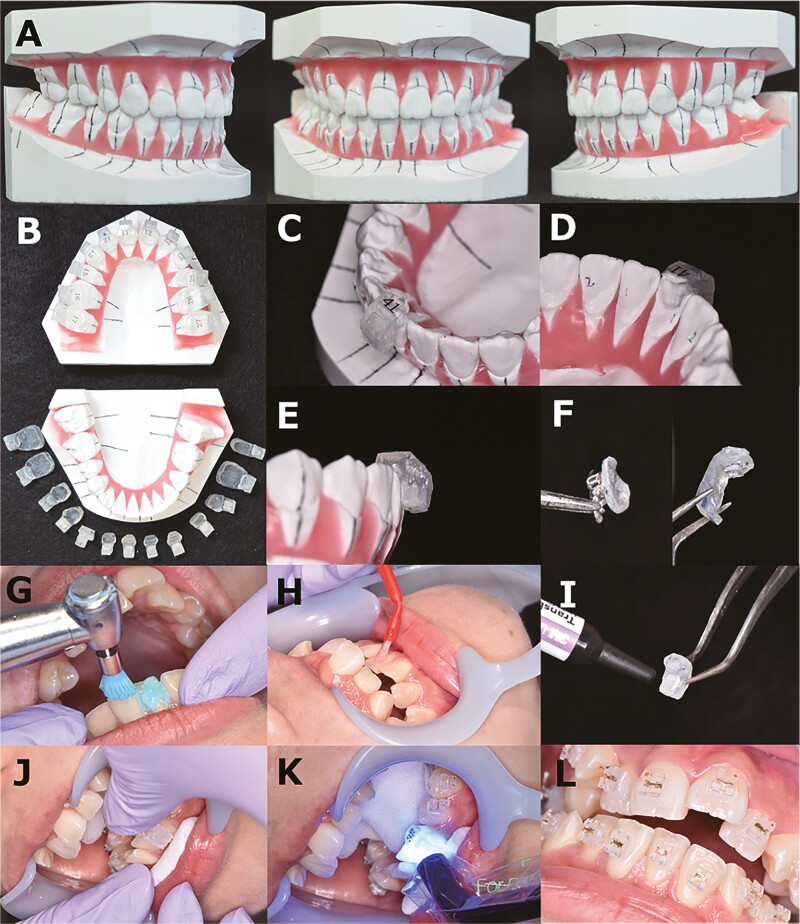
Indirect bonding technique. (A) a set-up model was created using the dental cast model taken at the initial visit. (B)The brackets are positioned on the set-up model. (C-E)The transfer tray was prepared for the transfer of indirectly placed brackets to the patient. (F)Base resin was attached to each bracket base. The base resin was made as thin as possible. (G)The teeth were cleaned with polishing brush. (H) Etching was applied to the tooth surfaces at the points of bracket placement. (I) Application of flowable composites to the sandblasted bracket bases. (J, K) After the transfer tray was placed, photopolymerization was achieved with a curing light device applied. (L) bracket was attached.

In the direct bonding group, after cleaning the teeth to remove plaque (Fig. 1A), the teeth were etched (Fig. 1B) using 40% phosphoric acid gel (KURARAY Co., Ltd., Tokyo, Japan) for 40 s. The samples were rinsed and dried with oil-free compressed air for 15 s. After drying the enamel surface, the liquid primer Transbond XT (3M Japan Limited, Tokyo, Japan) was applied using a small brush and spread with oil-free compressed air..018 slot Preadjusted plastic brackets (iPass II, ORTHO DENTAURUM, Tokyo, Japan) (Esther MB, TOMY INTERNATIONAL INC, Tokyo, Japan) or preadjusted metal brackets (Metal Bracket, JM ORTHO, Tokyo, Japan) were bonded using the Transbond XT Adhesive (3M Japan Limited). And irradiated for 3 s per bracket using the high-power mode or 10 s per bracket using the standard mode of curing light (PenCure 2000, MORITA, Kyoto, Japan), or irradiated for 10 s per bracket using the H1 mode of curing light (PEN Bright, SHOFU, Kyoto, Japan) (Fig. 1C–E). Excess resin was removed before light photopolymerization (Fig. 1F). In the indirect bonding group, a setup model was created using the dental cast model obtained at the initial visit (Fig. 2A). The bracket position was determined based on the setup model and the fabricated core (Fig. 2B–E). In this study, we used a single-tooth type core. Composite resin was attached to each bracket base (Fig. 2F). The brackets were positioned closer to the ideal arch, and the base resin was kept as thin as possible. At the chairside, the procedures for polishing (Fig. 2G), etching (Fig. 2H), and priming were the same as those for the direct bonding group. The adhesive was applied to the base surface of the bracket (Fig. 2I) and the core was pressed against the tooth surface (Fig. 2J). Excess resin was removed after the removal of the core and light was applied to cure the photopolymerization adhesive (Fig. 2K). The same bracket type was used in both techniques. Metal brackets were only used on upper and lower premolars when the crown length was short or there was no space for brackets in deep occlusion. Most cases had plastic brackets installed. Light photopolymerization was performed using a curing light in high-power mode or standard mode or H1 mode. Photopolymerization was repeated two to three times. The number of photopolymerization s was increased as necessary for the molars, which are difficult to reach with light. Indirect bonding was irradiated from above the tray, and direct bonding was irradiated from a position approximately 5 mm away from the bracket. The same procedure was used for both plastic brackets and metal brackets.

#### Follow-up

All enrolled patients will be followed up for 1–3 years after bonding. At the time of bonding, we will calculate the chair time (min) for each bonding procedure. Within a month of bonding, we will distribute a questionnaire to the patients, prompting them to describe their level of discomfort during the bonding procedure using a 100-point VAS. In addition, the oral hygiene index (%) will be calculated by a dental hygienist. After the treatment, the total treatment time (months), post-treatment occlusal index (scores), and failure rate (%) will be calculated (Supplementary Fig. 1). The post-treatment occlusal index of alignment, marginal ridges, buccolingual inclination, overjet, occlusal contacts, occlusal relationships, interproximal contacts, and root angulation were evaluated using CR-Eval. Model evaluation was performed blinding the indirect and direct bonding groups. The evaluation was performed by a doctor certified by the Japanese Orthodontic Society, and the second evaluation was performed 1 week after the first. The average of the two measurements was used as the occlusion index.

#### Randomization

After confirming the fulfilment of the eligibility criteria, the enrolled patients were randomized to either the direct or indirect bonding technique group. The project lead (K.K.), who was blinded to all clinical data, performed the allocation using medical record numbers.

### Statistical consideration

Outcomes were compared using a two-sample *t*-test or Mann–Whitney *U* test. The normality of the data was tested using the Shapiro-Wilk test. The *t*-test was completed on normally distributed data. Where data was non-parametric the Mann–Whitney *U* test was completed. Statistical significance was set at *P* < .05, at a confidence interval of 95%. All statistical analyses were performed using SPSS software (IBM Japan, Ltd., Tokyo, Japan).

## Results

### Participant flow (flow diagram, abandonment of treatment, and time periods)

**Figure 3.**
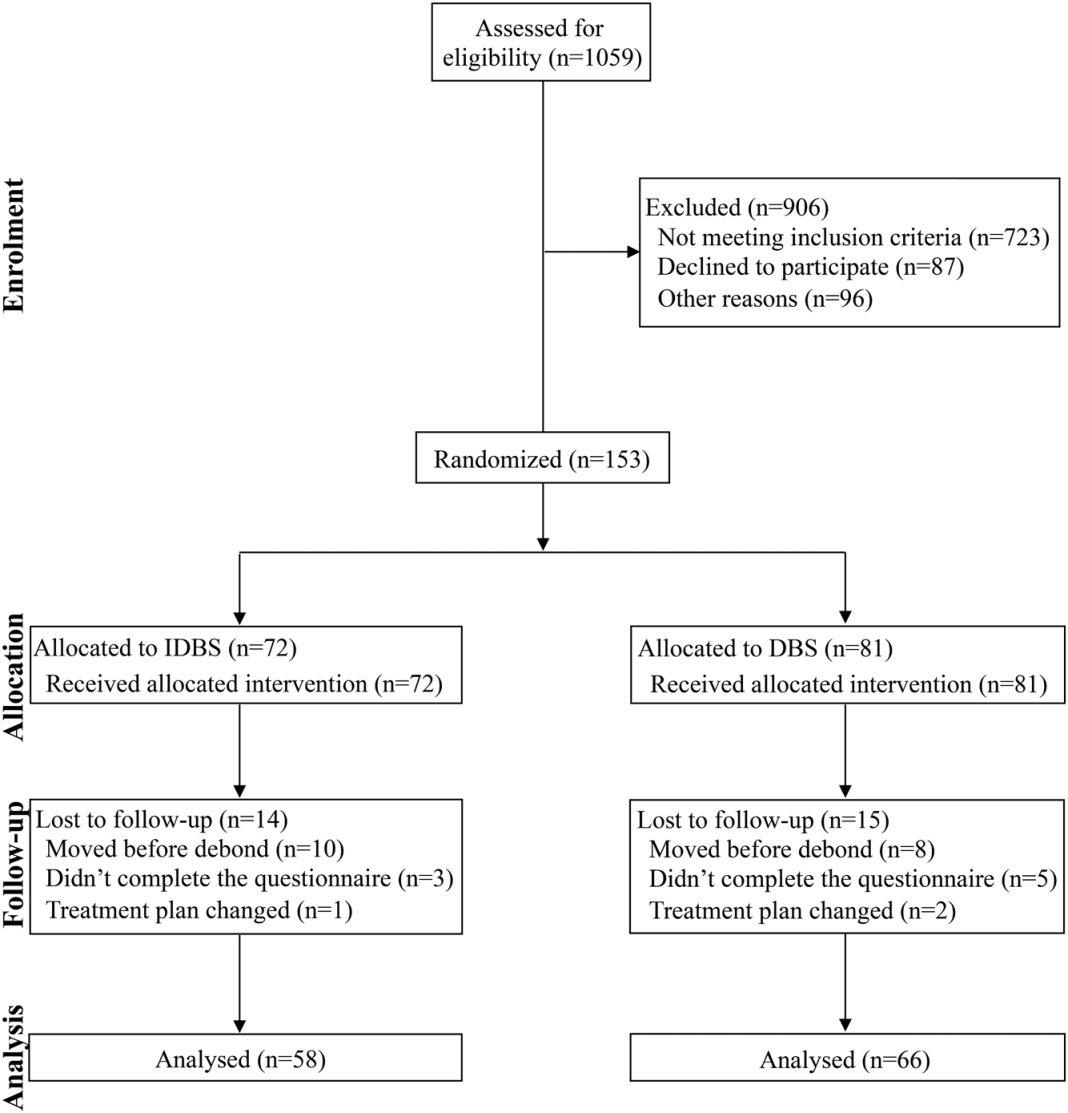
CONSORT diagram showing the flow of patients through the trial.

A total of 1059 patients who had been examined previously were recruited. Of these, 906 patients who did not meet the inclusion criteria or declined to participate were excluded before randomization (Fig. 3). A total of 153 patients were included for randomization. The patients were randomly divided into two groups. The indirect bonding group included 72 patients (23 male and 49 female) with a mean age of 20.63 ± 5.11 years. The direct bonding technique group included 81 patients (21 male and 60 female) with a mean age of 23.01 ± 7.92 years. During the study, 14 patients in the indirect bonding group and 15 patients in the direct bonding group were lost to follow up. Therefore, 124 patients were included in this study. Finally, 58 patients (20 male, 38 female) with a mean age of 20.63 ± 5.69 years were treated with the indirect bonding technique, and 66 patients (14 male, 52 female) with a mean age of 23.17 ± 8.83 years were treated with the direct bonding technique. The CONSORT chart showing the participant flow during this trial is shown in Fig. 3.

### Baseline data

Table 1 presents baseline data at the first visit. There was no significant difference in the cephalometric and cast analyses at the first visit between the indirect and direct bonding groups.

**Table 1.** Cephalometric analysis values and cast analysis values at the first visit of direct bonding (DBS) and indirect bonding (IDBS).

	DBS	IDBS	*P-valus*		DBS	IDBS	*P-valus*
**SNA(°)**	81.50 ± 2.87	82.52 ± 3.78	NS	**OJ(mm)**	4.35 ± 3.14	4.70 ± 2.24	NS
**SNB(°)**	77.48 ± 3.55	78.64 ± 4.27	NS	**OB(mm)**	2.45 ± 1.89	2.22 ± 1.98	NS
**ANB(°)**	5.92 ± 11.90	3.87 ± 2.62	NS	**U6/NF(mm)**	24.92 ± 2.51	24.70 ± 2.69	NS
**Mp-SN(°)**	36.27 ± 6.78	35.75 ± 6.32	NS	**U1/NF(mm)**	31.48 ± 3.73	30.87 ± 3.31	NS
**Mp-FH(°)**	28.53 ± 6.63	28.37 ± 6.37	NS	**L6/Mp(mm)**	35.58 ± 3.31	35.14 ± 3.12	NS
**Nf-Mp(°)**	27.61 ± 6.98	27.05 ± 6.12	NS	**L1/Mp(mm)**	46.80 ± 3.72	46.08 ± 3.88	NS
**U1-SN(°)**	107.87 ± 7.83	109.36 ± 9.59	NS	**L1/AP(mm)**	5.46 ± 3.44	5.48 ± 5.66	NS
**U1-FH(°)**	115.60 ± 7.63	116.66 ± 9.56	NS	**A.L.D(U)(mm)**	-3.97 ± 4.54	-2.16 ± 6.46	NS
**U1-NF(°)**	116.67 ± 7.58	117.85 ± 8.66	NS	**A.L.D(L)(mm)**	-3.82 ± 4.64	-2.93 ± 4.20	NS
**L1-FH(°)**	56.16 ± 10.48	56.49 ± 9.30	NS				
**L1-Mp(°)**	95.52 ± 9.09	94.16 ± 10.93	NS				
**L1-AP(°)**	26.60 ± 8.99	25.68 ± 5.74	NS				
**IIA(°)**	118.57 ± 17.00	121.37 ± 18.53	NS				
**Occp(°)**	18.40 ± 13.08	16.54 ± 4.87	NS				

### Chair time (min)

The time required for bonding and banding was measured as chair time. Bonding was defined to include tooth surface cleaning, curing, and excess resin removal. Banding was defined to include tooth surface cleaning, trial fitting, curing, and removal of excess cement.

**Figure 4.**
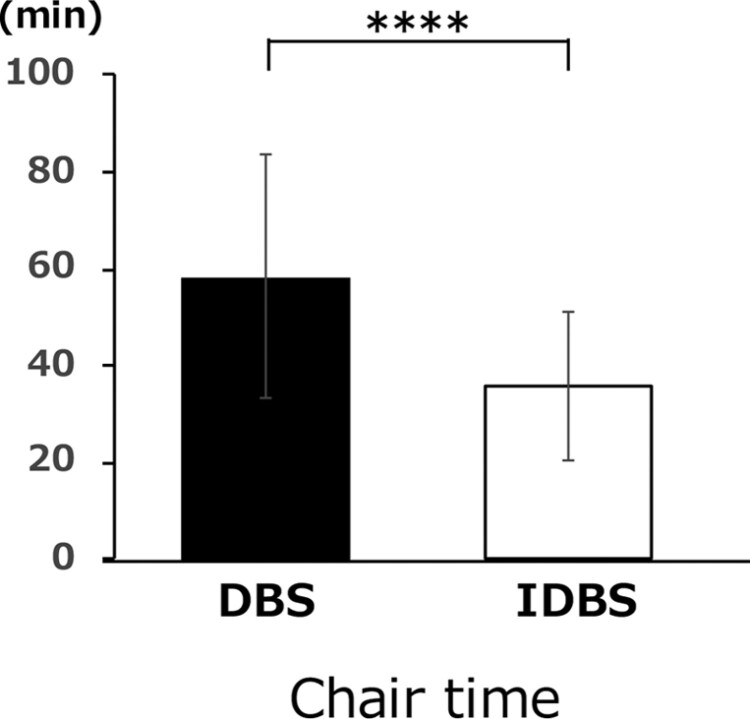
The chair time required for bracket attachment for IDBS and DBS. *P < .05, **P < .01, ***P < .005, ****P < .001.

Although there were variations in the data, the time required to bond brackets from mandibular incisors to the first molars was significantly shorter in the indirect bonding group (indirect bonding group, 35.91 ± 15.51 min; direct bonding group, 58.52 ± 25.24 min) (Fig. 4).

### Discomfort at bonding (VAS scores, mm)

The questionnaire evaluated the discomfort during bonding for the anterior teeth (mandibular incisors to the premolars) and mandibular first molars (Table 2).

**Table 2.** Discomfort at bonding.

Questions			
Q1. Did you feel uncomfortable while putting on the device?			
Q2. Did it take a long time to attach the device?			
Q3. Did you feel any discomfort after attaching the device?			

Results (VAS scores: mm)			
Anterior teeth (mandibular incisors premolars)			
	**DBS**	**IDBS**	***P-valus***
Q1	25.69 ± 18.23	26.31 ± 17.48	NS
Q2	29.26 ± 18.52	31.18 ± 19.84	NS
Q3	47.10 ± 21.09	52.09 ± 23.08	NS

Mandibular first molars			
	**DBS**	**IDBS**	***P-valus***
Q1	31.43 ± 20.93	31.12 ± 19.50	NS
Q2	29.72 ± 21.88	29.51 ± 21.04	NS
Q3	45.81 ± 24.27	44.47 ± 24.50	NS

A 100-point visual analogue scale (VAS) was used to assess discomfort during the bonding procedure. *P* < .05, NS: not significant.

There was no significant difference in the discomfort at bonding between the anterior teeth (mandibular incisors to premolars) and mandibular first molars (Table 2).

### Oral hygiene (PCR: plaque control record, %)

**Figure 5.**
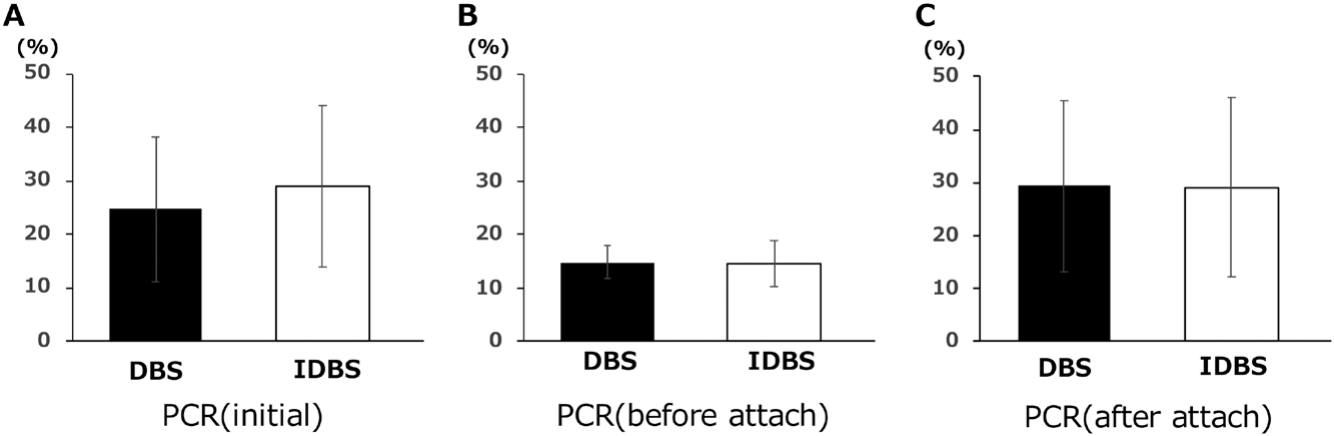
Oral hygiene (PCR: plaque control record). (A) PCR in indirect bonding (IDBS) and direct bonding (DBS) at initial. (B) PCR in IDBS and DBS after oral hygiene instruction. (C) PCR in IDBS and DBS after bracket placement. *P < .05, **P < .01, ***P < .005, ****P < .001.

The initial PCR was 29.04 ± 15.11% for the indirect bonding group and 24.76 ± 13.53% for the direct bonding group (Fig. 5A). The PCR before bracket attachment was 14.55 ± 4.23% in the indirect bonding group and 14.79 ± 3.02% in the direct bonding group (Fig. 5B). There were no differences in the PCR results between the two groups.

The PCR after bracket attachment was 29.08 ± 16.84% in the indirect bonding group and 29.38 ± 16.13% in the direct bonding group (Fig. 5C). There was no difference in the PCR results between the two groups.

### Number of bracket failures (pieces)

**Figure 6.**
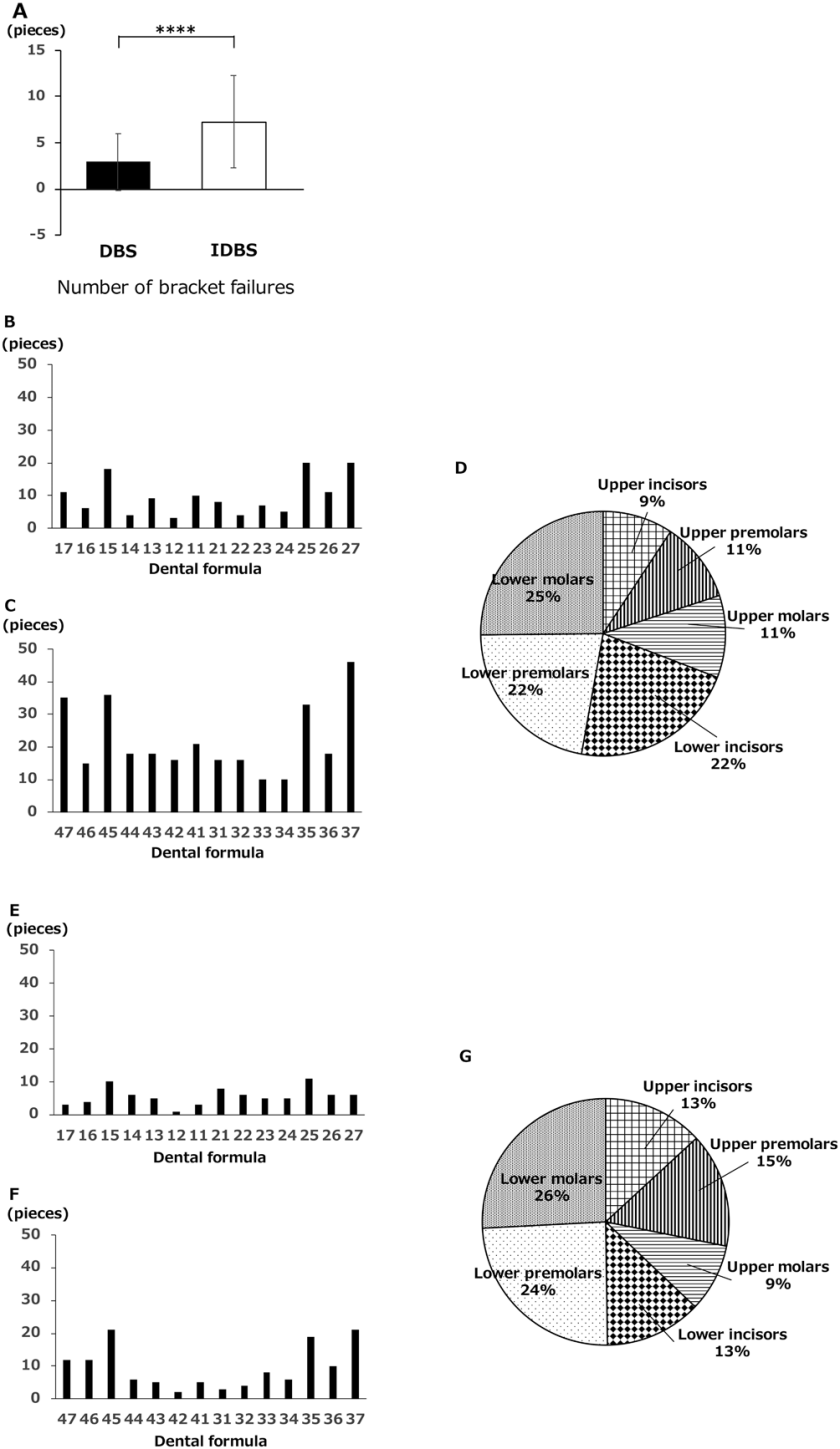
The number of bracket failures (bracket detachment from a tooth). (A) Failure (bracket detachment from a tooth) number in indirect bonfing (IDBS) and direct bonding (DBS). (B) Comparison of bracket failure sites in maxillary dentition in IDBS. (C) Comparison of bracket failure sites in mandibular dentition in IDBS. (D) Comparison of bracket failure sites in IDBS. (E) Comparison of bracket failure sites in maxillary dentition in DBS. (F) Comparison of bracket failure sites in mandibular dentition in DBS. (G) Comparison of bracket failure sites in DBS. *P < .05, **P < .01, ***P < .005, ****P < .001.

Although there were variations in the data, the number of bracket failures per case was significantly higher in the indirect bonding group (7.28 ± 4.98) than in the direct bonding group (2.92 ± 3.07) (Fig. 6A).

The number of failures in the indirect bonding group was generally higher than that in the direct bonding group, with mandibular teeth failure being higher overall and tooth 37 having a particularly high failure rate (Fig. 6B–D).

The direct bonding group showed a greater number of failures in the mandibular premolar and molar regions. Of these, teeth 45 and 37 failed most frequently (Fig. 6E–G).

Metal brackets accounted for 8.61% (indirect bonding group) and 8.49% (direct bonding group) of all brackets. There was no difference between the two groups.

The bracket failure rate for upper and lower premolars in the indirect bonding group was 11.35% for metal brackets and 37.70% for plastic brackets. When divided into upper and lower dentition, the metal bracket was 12.9% in the upper dentition, and 10.13% in the lower j dentition, the plastic bracket was 24.8% in the upper dentition, 42.5% in the lower dentition. Significant differences were observed in the lower dentition.

The bracket failure rate for upper and lower premolars in the direct bonding group was 11.04% for metal brackets and 22.84% for plastic brackets. When divided into upper and lower dentition, the metal bracket was 10% for the upper dentition and 11.38% for the lower dentition, plastic bracket was 18.79% for the upper dentition, 29.23% for the lower dentition.

### Number of intentional reattachments (pieces)

Intentional reattachment was performed when it was difficult to improve with wire bending alone.

**Figure 7.**
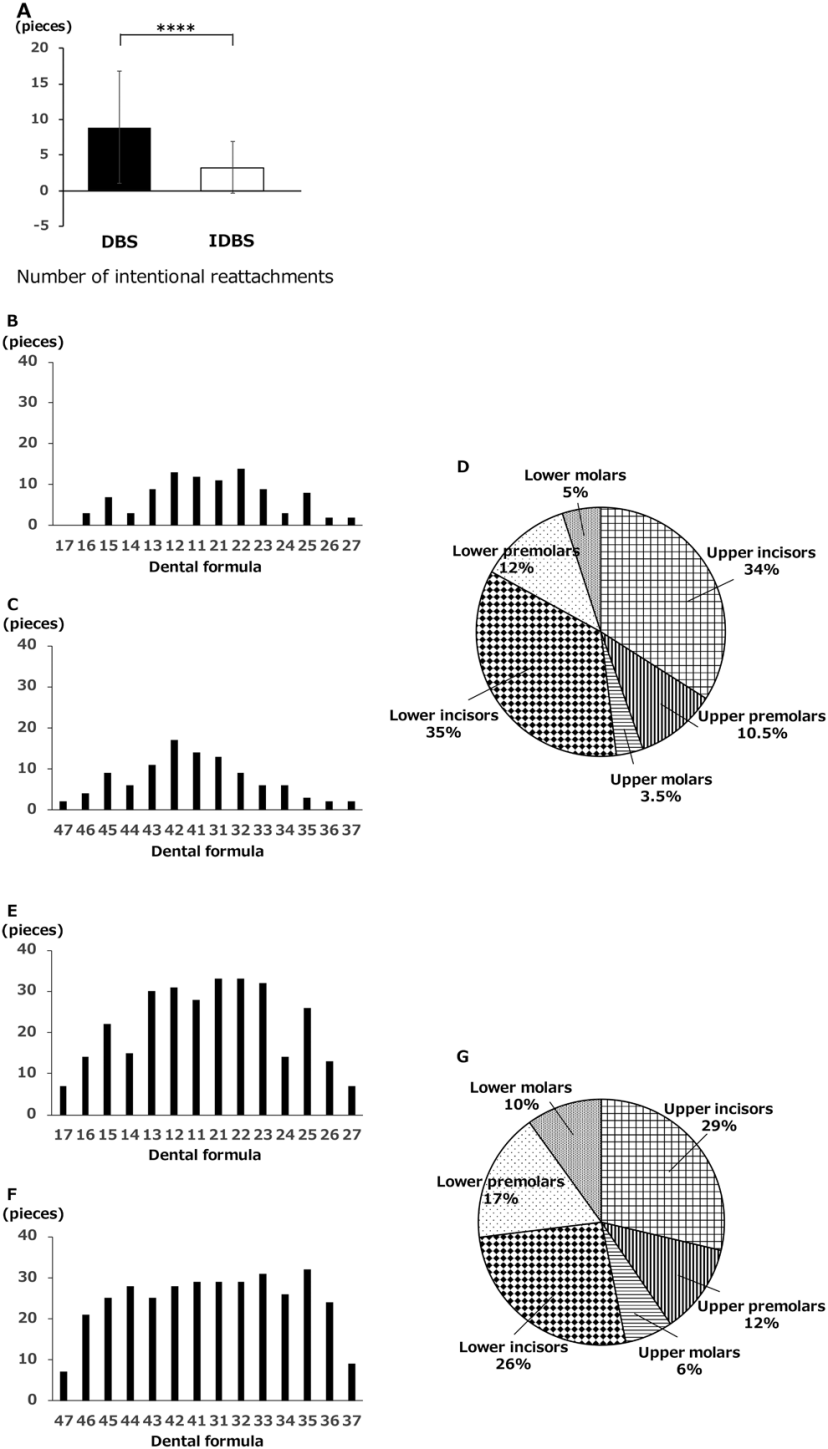
The number of intentional reattachments. (A) The number of intentional reattachment in indirect bonding (IDBS) and direct bonding (DBS). (B) Comparison of bracket intentional reattachment sites in maxillary dentition in IDBS. (C) Comparison of intentional reattachment sites in mandibular dentition in IDBS. (D) Comparison of bracket intentional reattachment sites in IDBS. (E) Comparison of bracket intentional reattachment sites in maxillary dentition in DBS. (F) Comparison of bracket intentional reattachment sites in mandibular dentition in DBS. (G) Comparison of bracket intentional reattachment sites in DBS. *P < .05, **P < .01, ***P < .005, ****P < .001.

The number of intentional reattachments per case was significantly lower in the indirect bonding group (3.26 ± 3.59) than that in the direct bonding group (8.89 ± 7.82) (Fig. 7A).

In the direct bonding group, the number of intentional reattachments was high in the maxillary anterior teeth, especially in teeth 21 and 22. Compared to the direct bonding group, the number of intentional reattachments was lower overall in the indirect bonding group but higher in the mandibular anterior teeth, particularly for tooth 42 (Fig. 7B–G).

### Post-treatment occlusal index (CR-Eval, scores)

**Figure 8.**
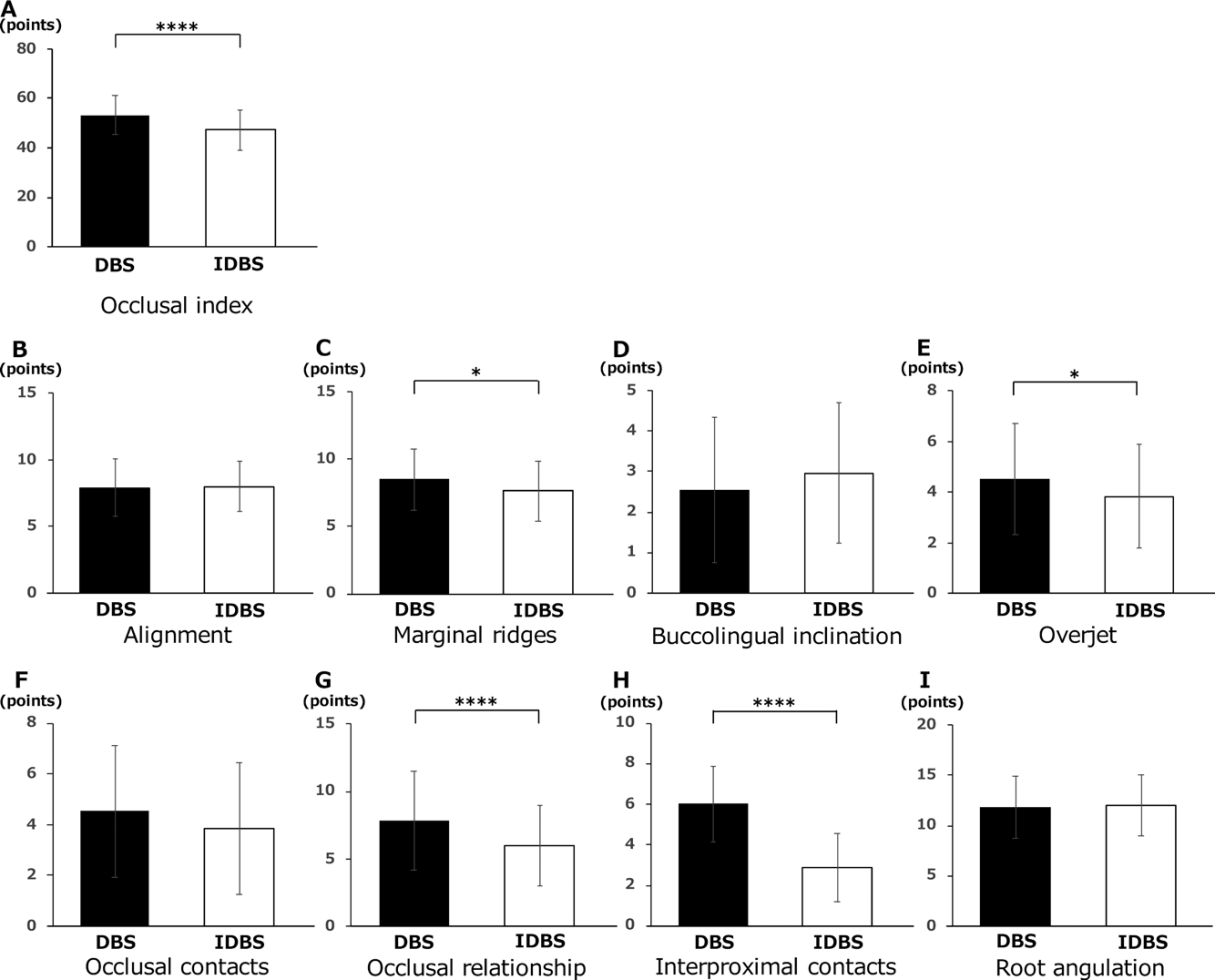
Occlusal index (CR-Eval). (A) The occlusal index were evaluated using the ABO-Scoring index (CR-Eval). (B) Alignment (C) marginal ridges (D) buccolingual inclination (E) overjet (F) occlusal contacts (G) occlusal relationships (H) interproximal contacts (I) root angulation. *P < .05, **P < .01, ***P < .005, ****P < .001.

Although there were variations in the data, the occlusal relationship score was significantly lower in the indirect bonding group (47.31 ± 8.17) than in the direct bonding group (53.24 ± 7.74) (Fig. 8A).

No significant difference was observed in alignment between the indirect bonding (8.01 ± 1.91) and direct bonding (7.89 ± 2.14) groups (Fig. 8B). Marginal ridges were significantly better in the indirect bonding group (7.62 ± 2.25) than those in the direct bonding group (8.51 ± 2.26) (Fig. 8C). No significant difference was observed in the buccolingual inclination between the indirect (2.96 ± 1.73) and direct (2.54 ± 1.79) bonding groups (Fig. 8D). Overjet was significantly better in the indirect bonding group (3.84 ± 2.05) than in the direct bonding group (4.54 ± 2.20) (Fig. 8E). No significant difference was observed in the occlusal contacts between the indirect (4.04 ± 2.59) and direct (4.36 ± 2.59) bonding groups (Fig. 8F). Occlusal relationship was significantly better in the indirect bonding group (6.00 ± 2.95) than in the direct bonding group (7.85 ± 3.64) (Fig. 8G). Interproximal contacts were significantly better in the indirect bonding group (2.87 ± 1.67) than in the direct bonding group (6.01 ± 1.89) (Fig. 8H). No significant difference was observed in the root angulation between the indirect (11.97 ± 3.05) and direct (11.84 ± 3.12) bonding groups (Fig. 8I).

The intraclass correlation coefficient (ICC) was calculated to assess intrarater reliability. ICC (1,1) was 0.953 (indirect binding group) and 0.857 (direct binding group), which was greater than 0.75, so the measurements are considered to be sufficiently consistent and reproducible.

### Total treatment period (months)

**Figure 9.**
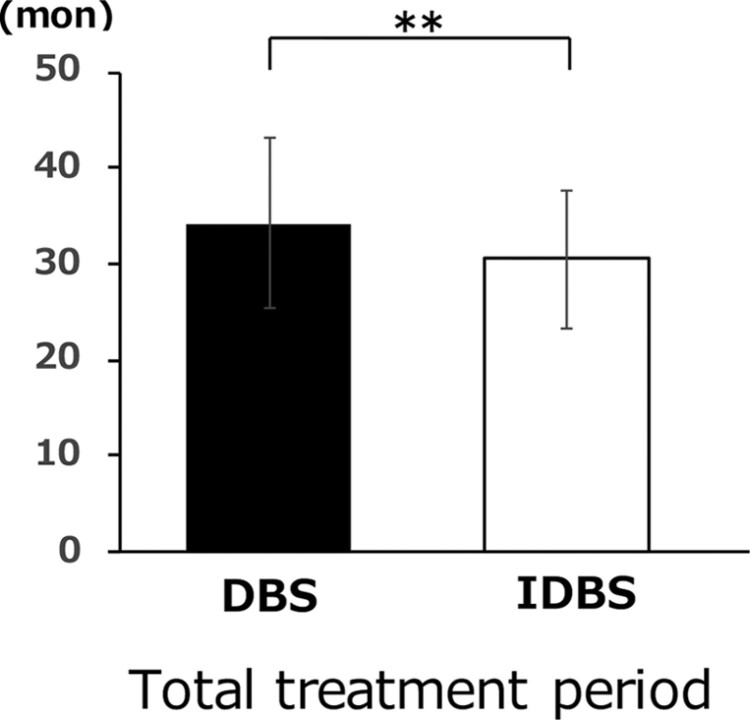
Total treatment period (months). The treatment period required from brackets attached to removed in indirect bonding (IDBS) and direct bonding (DBS). *P < .05, **P < .01, ***P < .005, ****P < .001.

The total treatment period was significantly shorter in the indirect bonding group (30.51 ± 7.27 months) than in the direct bonding group (34.27 ± 8.87 months) (Fig. 9).

### Harms

No harms were observed during the study.

## Discussion

### The importance of the study

The advantages and disadvantages of direct bonding and indirect bonding have been much discussed [7–10].However, most reports compared and evaluated single factors involved in direct and indirect methods, and few prospective studies have considered the relative effects of all factors. Therefore, this study is the first to evaluate and compare the direct and indirect methods through comprehensive and detailed clinical evaluation at a single institution and to clarify the selection criteria for the bonding method.

### Interpretation of the findings and comparisons with previous studied

#### Baseline data

Linear and angular measurements were performed using cephalometric at the first visit. As a result, there were no significant differences between the indirect and direct bonding groups in both skeletal and dental outcomes. In addition, we performed cast analyses at the first visit. As a result, there was no significant difference in the arch length discrepancy between the indirect and direct bonding groups. Based on these findings, it seems that there was no difference between indirect and direct bonding groups regarding the degree of malocclusion at the time of initial examination.

#### Chair time

A previous study showed that one of the advantages of indirect bonding is the reduction in bonding time [19]. This technique does not require further time to decide the position of the brackets during bonding [20]. A study comparing the time spent on indirect and direct bonding of both arches (including all molar teeth) showed that the total time spent on bonding was reduced by 30 min during indirect bonding [21]. This study also showed that the bonding time for indirect bonding was significantly shorter than that for direct bonding. However, previous reports have mentioned that the time required to complete the indirect bonding procedure, including the time in the laboratory, is significantly longer than that required to complete the direct bonding procedure [12]. However, as greater digitalization is expected in the future, it may enable lesser laboratory time for the indirect bonding procedure.

#### Discomfort at bonding

No significant difference was observed between indirect and direct bonding in terms of discomfort during bonding, either from the incisors to the premolars or in the first molars. This was similar to the results of a previous study that assessed discomfort during the banding and bonding of the first molars [22]. The core used when attaching the brackets does not cause significant discomfort. Although direct bonding increases the chair time, it is believed that longer chair time may cause less discomfort. However, although it was clear that there was no difference in discomfort at bonding between the direct bonding and indirect bonding, it is possible that the questionnaire was performed within a month after bonding rather than immediately after bonding, which may have introduced a response shift. It is possible that patients adapted psychologically to better cope with the new life circumstances, reflecting better outcomes over time. Originally this risk should have been eliminated by performing the questionnaire at bonding.

#### Oral hygiene

No significant difference was observed in PCR after attaching brackets between indirect and direct bonding. During the first visit, the patient was guided by a dental hygienist. After confirming that the PCR was 20% or less, the operator attached the bracket. One month after bracket attachment, PCR was measured again. Previous studies have reported no significant differences between indirect and direct bonding in terms of plaque accumulation around brackets, formation of white spot lesions [14, 23], and gingival conditions [11, 14]. Similar results were obtained in this study.

#### Number of bracket failures

Previous clinical trials comparing the direct and indirect methods have reported failure rates of 2% for direct bonding and 13% for indirect bonding [11]. Another study reported a failure rate of 35% for indirect bonding [4]. In this study, the failure rate was 20.10% for indirect bonding and 7.75% for direct bonding. The number of bracket failures per case was 7.28 ± 4.98 for indirect bonding and 2.92 ± 3.07 for direct bonding (Fig. 6A). The results of this study are consistent with those of previous studies, although one of the reasons for the slightly higher failure rate may be that the operators were less experienced rather than experienced operators. Additionally, the number of failures in indirect bonding was generally higher than that in direct bonding. Possible reasons for this include insufficient moisture proofing and insufficient photocuring. The number of failures in indirect bonding was generally higher than that in direct bonding, with the mandibular teeth having higher overall failure and tooth 37 having a particularly high failure rate (Fig. 6B–D). Direct bonding resulted in a greater number of failures in the mandibular premolar and molar regions. Of these, teeth 45 and 37 failed most frequently (Fig. 6E–G). A previous study investigating the failure rate of indirect bonding found no significant difference in the failure rates of both the upper and lower arch attachments. Similarly, no significant difference was found between the anterior and posterior failure rates when data from both arches were analysed individually or in combination [4]. In this study, the number of failures was significantly higher in the lower arch than in the upper arch for both indirect (*P* > .001) and direct bonding (*P* > .05). Furthermore, bracket failure in the upper arch did not differ significantly between the anterior and posterior areas for either indirect or direct bonding. In the lower arch, only direct bonding resulted in a significantly higher number of posterior failures (*P* > .01). Moreover, comparing the upper and lower arches, direct bonding was significantly higher in the posterior region (*P* > .001), while indirect bonding was not significantly different between the anterior and posterior regions. However, in a previous study, a higher posterior failure rate was detected in posterior teeth [24]. One possible explanation for the high posterior failure rate is the increased difficulty in isolation. Furthermore, occlusal interference may result in high bracket failure in the mandibular dentition. In addition, in indirect bonding, the base resin intervenes between the tooth surface and the bracket base, making pressure application more difficult than in direct bonding. Moreover, in indirect bonding, the bracket is placed in an ideal position; therefore, compared with direct bonding, where the bracket position can be adjusted according to the treatment stage, the bracket may come off more easily during levelling.

Also, in this study, two types of brackets were used only for the upper and lower premolars. Although differences in failure rates were observed depending on the type of bracket, in both cases, the failure rate was found to be significantly higher in the indirect bonding group.

Regarding bracket type, the failure rate of plastic brackets in the lower dentition was significantly higher in the indirect bonding group. It has been reported that one of the reasons is that plastic brackets are inferior in strength and durability and have weak shear bond strength compared to metal brackets [25]. Although the performance has improved compared to conventional products, it is thought that in areas such as the lower dentition, where large forces are applied to occlusion, it may have been easier to come off due to occlusal interference. Furthermore, because plastic brackets are larger than metal brackets, interference with occlusion is more likely to occur, which may also be a factor in the high rate of failure.

Next, the Kaplan–Meier survival graph is shown in Supplementary Fig. 2. The survival curves based on the first failure time for each bonding method were significantly different between the indirect bonding group and the direct bonding group (*P* < .001). The period until the bracket first failure was shorter in the indirect bonding group, and in the indirect bonding group, there were many cases of bracket failure early after attachment. This result agrees with previous reports that most bracket failures occur within 3 months after attachment [26]. One of the reasons for failure may be an inappropriate bonding method. On the other hand, although there was a certain number of initial failures in the direct bonding group, the survival curve was gentler than that in the indirect bonding group, and there were many cases other than initial failures. Therefore, it was suggested that patient factors such as habit, diet, and trauma may be the cause.

#### Number of intentional reattachments

Furthermore, we evaluated the number of intentional reattachments of brackets. The intentional reattachment rate was 8.73% for indirect bonding and 24.39% for direct bonding, with a significantly higher rate for direct bonding. The number of intentional reattachments per case was significantly lower in indirect bonding (3.26 ± 3.59) than in direct bonding (8.89 ± 7.82) (Fig. 7A). Regarding the accuracy of bracket placement position, indirect bonding has been reported to be superior because it is easier to attach brackets to the model than to attach them directly to the teeth *in vivo* (better visual acuity, unlimited work time) [3, 7, 10]. Based on these factors, indirect bonding had a better bracket positioning accuracy than direct bonding; therefore, it is possible that reattachment of bracket to a better position was not required. Furthermore, the number of intentional reattachments did not differ significantly between the upper and lower arches. However, when comparing the anterior and posterior arches, the upper arch had a significantly higher anterior intentional reattachment in both indirect (*P* > .001) and direct bonding (*P* > .001). In the lower arch, only indirect bonding resulted in a higher number of anterior reattachments (*P* > .001). Moreover, when comparing the upper and lower arches together, both indirect (*P* > .001) and direct bonding (*P* > .001) had a significantly higher number of anterior attachments. By site, the number of intentional reattachments was higher in the upper anterior teeth during direct bonding, particularly in teeth 21 and 22. Compared to direct bonding, the number of intentional reattachments was lower overall in indirect bonding but higher in the lower anterior teeth, particularly tooth 42 (Fig. 7B–G). The reason for the large number of intentional reattachments in the anterior teeth may be that frequent bracket reattachments are necessary to achieve better alignment, as the area is directly related to aesthetics.

#### Post-treatment occlusal index

Occlusal relationship scores were significantly lower for indirect bonding. The occlusal relationship at the end of the treatment was significantly better for indirect bonding. In a previous study that investigated CR-Eval at the end of treatment, 54.9% of cases had a score of 30 or more [27]; thus, the scores obtained in this study were reasonable. However, the reliability of CR-Eval is lower than expected, and some studies suggest that it may still be overly subjective [28]. Therefore, the results of this study can be considered strict, with slightly higher overall scores. In addition, one of the reasons why the overall treatment time was slightly longer, and the CR-Eval score was higher, may be that the operators were postgraduate residents rather than experienced operators and had fewer years of experience. However, it must be kept in mind that operators have little experience with both Techniques. Although experienced practitioners may have extensive experience with one or the other technique, our operators are likely to have similar experience with both techniques.

The results of this study suggest that indirect bonding has a higher bracket position accuracy than direct bonding, which is a factor in the significantly better occlusal relationship in indirect bonding. Furthermore, alignment, marginal ridges, buccolingual inclination, overjet, occlusal contacts, occlusal relationships, interproximal contacts, and root angulation were evaluated using the CR-Eval. Indirect bonding yielded significantly better results for marginal ridges, overjet, occlusal relationships, and interproximal contacts. In direct bonding, a band is often attached to the molar, and the band space remains immediately after debonding; therefore, interproximal contact is considered to have a significantly higher score in direct bonding.

#### Total treatment period

The total treatment period was significantly shorter for indirect bonding. A previous study reported no significant difference in the total treatment time between the two groups, and the number of appointments did not differ between the two techniques [13]. However, one advantage of indirect bonding is its positional accuracy. The total treatment time may have shortened because the brackets could be attached in a more ideal position. In addition, computer-assisted bracket placement systems pursued by several companies claim to provide accurate bracket placement and significantly reduced treatment time [29]. The accuracy of the setup model and core of the indirect method used in this study were high, and the bracket position was considered ideal.

### Limitations

The absence of patients’ blinding could not affect the results, since laypeople should not have a more favourable attitude toward one technique or the other, both being totally unknown to them. However, the absence of the operator’s blinding could influence his or her impartiality and generate a bias by favouring one technique during the bonding procedure. However, since blinding was not possible, the operator attempted to work with a neutral attitude toward both techniques.

The randomization allocation method was based on medical record numbers, which could induce selection bias due to the high predictability of allocation, making it a quasi-randomized controlled trial. Selection bias should have been eliminated by using a random number table. However, no significant differences were observed between the two groups in patients’ baseline data.

Because plastic and metal brackets were used, it remains questionable in our study whether the results actually reflect differences in bonding methods or are related to differences in bracket type. However, since there was no significant difference in the percentage of all brackets placed on premolars between plastic and metal brackets, it can be assumed that the difference is related to the difference in the bonding method.

Intentional reattachment was performed when the bracket failed or when it was difficult to improve with wire bending alone. However, because there are individual differences in wire bending techniques and preferences, it is possible that bias between operators occurred. However, since the operators were the same in both the IDBS and DBS groups, comparisons between the two groups are considered possible.

### Generalizability

Because the brackets were bonded by postgraduate residents, the results of this study were influenced by a learning process, and the generalizability of the results obtained to the reality of private practice with an experienced operator might be limited. However, it must be kept in mind that the operators had little experience with both Techniques. Experienced operators may find indirect bonding to be fast and have better results, but direct bonding is probably just as fast and good.

## Conclusions

The results of our study demonstrated the efficacy of the indirect bonding technique in comparison to those of the direct bonding technique. Orthodontists can select one technique based on chair time, laboratory time, patient acceptance, quality of the results, cost, or personal preferences.

However, it has been suggested that for orthodontist with less years of clinical experience, using indirect bonding techniques is more advantageous in shortening treatment time and treatment period, and in achieving good occlusal relationships after treatment. Although, in the case of indirect bonding method, the failure rate was particularly high in the mandibular molar region, so if it is difficult to apply pressure to the tray, or if it is difficult to moisture proof or light irradiate the molar region, it is recommended to use direct bonding method. Furthermore, although it is less aesthetically pleasing, it is recommended to use metal brackets for the mandibular molars.

## Supplementary Material

cjae036_suppl_Supplementary_Figure_S1

cjae036_suppl_Supplementary_Figure_S2

cjae036_suppl_Supplementary_Table_S1

## Data Availability

The data underlying this article will be shared on reasonable request to the corresponding author.
