# Supplementary figures and images for: Comprehensive clinical evaluation of indirect and direct bonding techniques in orthodontic treatment: a single-centre, open-label, quasi-randomized controlled clinical trial
Source: Eur J Orthod. 2024 Oct 4;46(6):cjae036. doi: 10.1093/ejo/cjae036 (PMC11450403; doi:10.1093/ejo/cjae036)

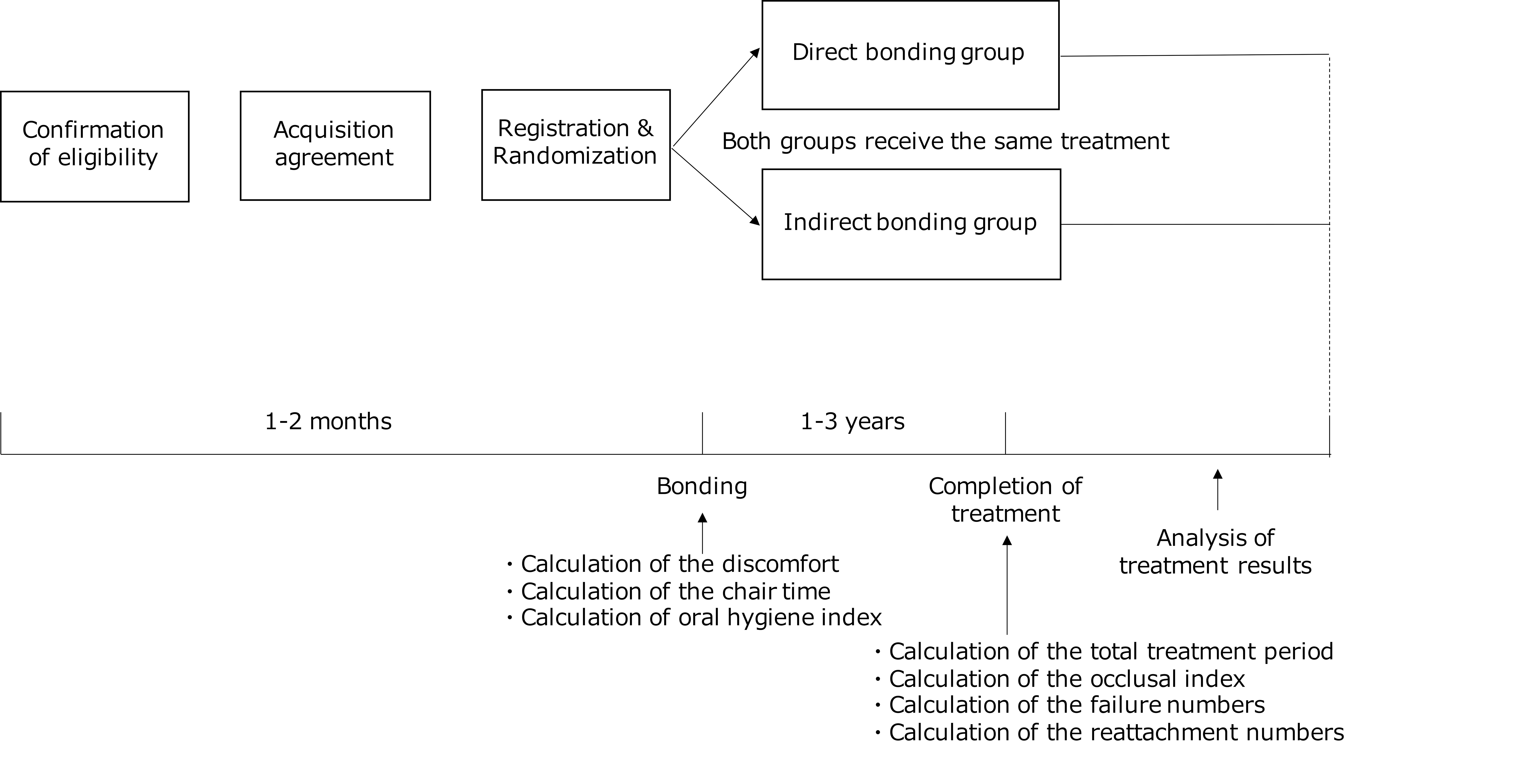

Supplement: cjae036_suppl_Supplementary_Figure_S1 [file cjae036_suppl_supplementary_figure_s1.jpeg]

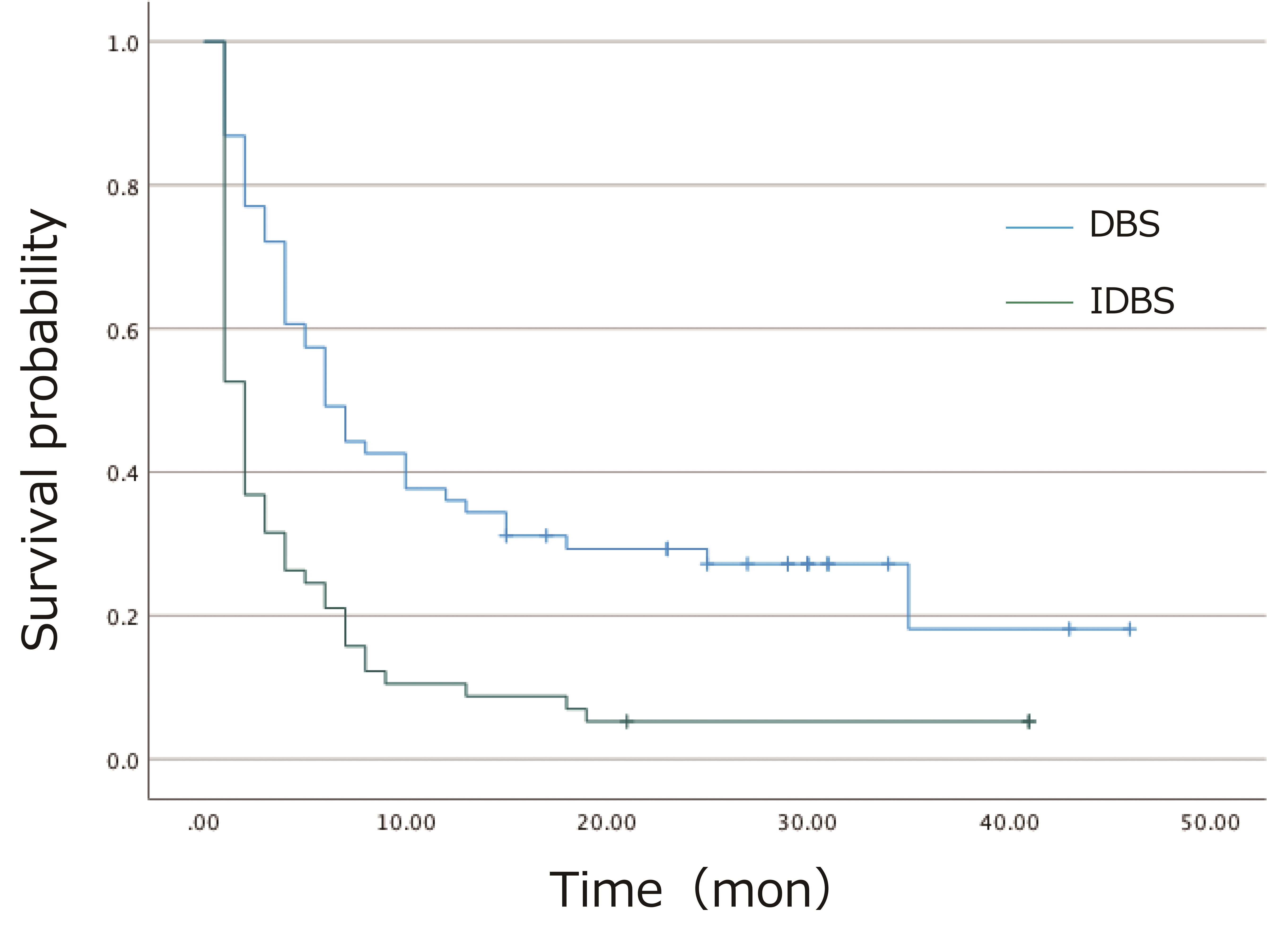

Supplement: cjae036_suppl_Supplementary_Figure_S2 [file cjae036_suppl_supplementary_figure_s2.jpeg]

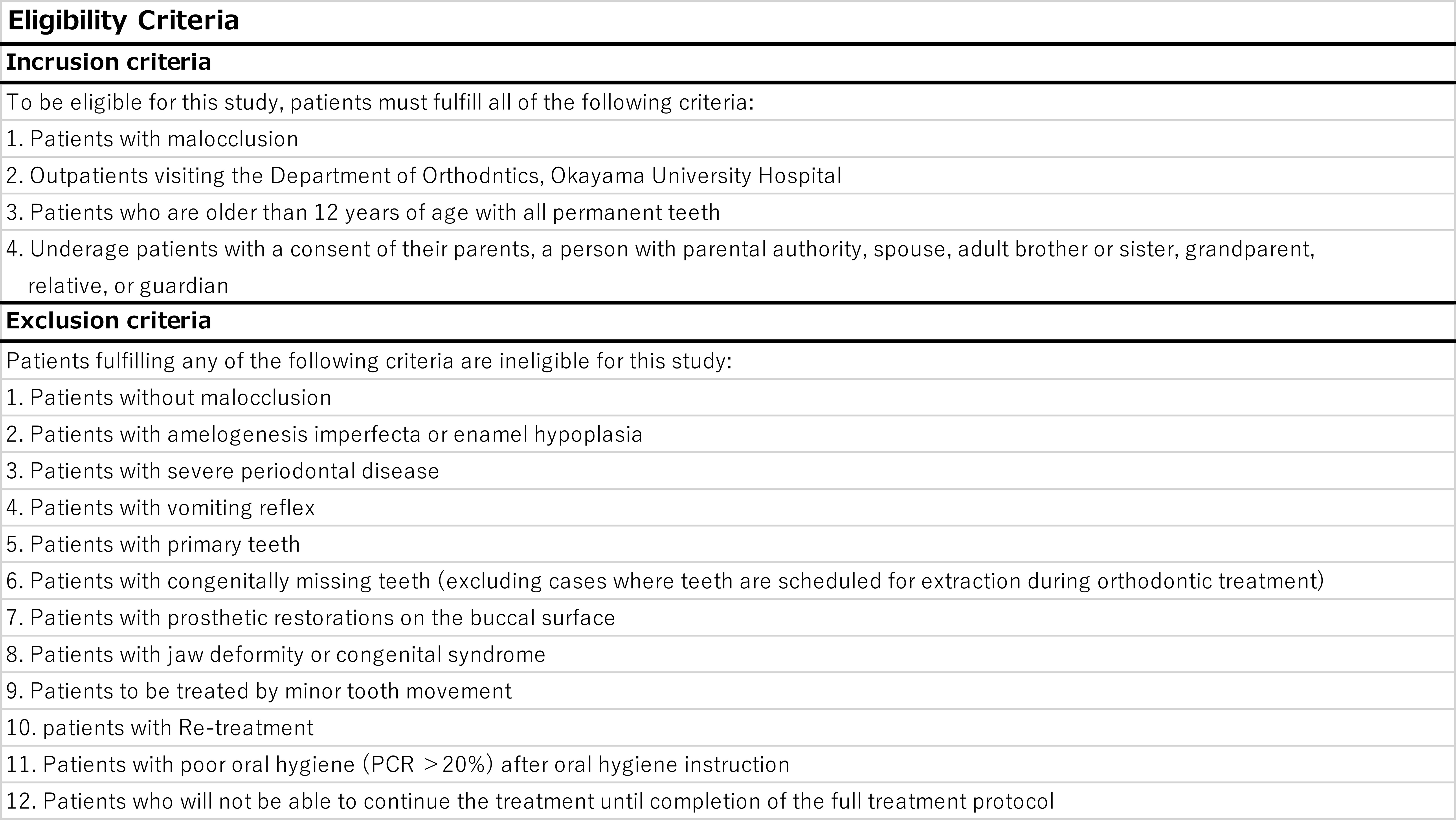

Supplement: cjae036_suppl_Supplementary_Table_S1 [file cjae036_suppl_supplementary_table_s1.jpeg]
